# Traumatic Brain Injury in 2020 – a horizon-scanning project

**DOI:** 10.1186/1757-7241-22-S1-P6

**Published:** 2014-07-07

**Authors:** Neil Roberts, Domhnall O’Dochartaigh, Dawid Aleksandrowicz, Dean Whiting

**Affiliations:** 1 Queen Mary’s College, University of London, London, United Kingdom

## Background

Up to 62% of severe injuries in British patients include Traumatic Brain Injury (TBI) [1]. 48% of these severe TBIs have unfavourable 6-month outcome with large variations in mortality and morbidity between centres [2]. Most deaths occur from brainstem herniation [3]. This project aimed to evaluate current best-practice for gaps in knowledge, care, and research, to determine what changes could best improve TBI care, and to project optimum treatment for 2020.

## Methods

Post-graduate students constructed a current optimal patient pathway following brainstorming exercises. A comprehensive literature review of current and promising new treatments was performed. A new care pathway based on the direction of current research was predicted for 2020, which we believe could improve outcomes.

## Results

Best-practice TBI care is expensive and complex, requiring extensive infrastructure at all stages. Overall, there is weak evidence for much of TBI care. There are several reasons for this. TBI is often addressed as a single entity, rather than individual injury patterns with variable pathophysiology. Monitoring in the acute setting is difficult, with multiple options but no single modality with consistent evidence of benefit. Long-term outcome assessment is expensive with common tools providing little detailed information.

## Conclusions

Large improvements in TBI care can be made by consistently and effectively applying treatments with known benefits. Funding is required to secure infrastructure required for optimal TBI care and evidence is required to secure this. A shift in thinking from ‘TBI’ to the assessment and treatment of individual injury patterns is important. Multimodal monitoring with computerised analysis of large registries shows promise in guiding treatment and research. Functional imaging and Patient-Reported Outcome Measurements can facilitate better evidence for aftercare. Research into tools such as novel biomarkers or drugs is important long-term, but a higher-quality evidence base is needed first as a foundation towards minimising gaps in care.
